# Fast Interleaved Multislice T1 Mapping: Model-Based Reconstruction of Single-Shot Inversion-Recovery Radial FLASH

**DOI:** 10.1155/2018/2560964

**Published:** 2018-08-13

**Authors:** Xiaoqing Wang, Dirk Voit, Volkert Roeloffs, Martin Uecker, Jens Frahm

**Affiliations:** ^1^Department of Diagnostic and Interventional Radiology, University Medical Center, Göttingen, Germany; ^2^Biomedizinische NMR Forschungs GmbH, Max-Planck-Institut für biophysikalische Chemie, 37070 Göttingen, Germany; ^3^DZHK (German Centre for Cardiovascular Research), Partner Site, Göttingen, Germany

## Abstract

**Purpose:**

To develop a high-speed multislice T1 mapping method based on a single-shot inversion-recovery (IR) radial FLASH acquisition and a regularized model-based reconstruction.

**Methods:**

Multislice radial k-space data are continuously acquired after a single nonselective inversion pulse using a golden-angle sampling scheme in a spoke-interleaved manner with optimized flip angles. Parameter maps and coil sensitivities of each slice are estimated directly from highly undersampled radial k-space data using a model-based nonlinear inverse reconstruction in conjunction with joint sparsity constraints. The performance of the method has been validated using a numerical and experimental T1 phantom as well as demonstrated for studies of the human brain and liver at 3T.

**Results:**

The proposed method allows for 7 simultaneous T1 maps of the brain at 0.5 × 0.5 × 4 mm^3^ resolution within a single IR experiment of 4 s duration. Phantom studies confirm similar accuracy and precision as obtained for a single-slice acquisition. For abdominal applications, the proposed method yields three simultaneous T1 maps at 1.25 × 1.25 × 6 mm^3^ resolution within a 4 s breath hold.

**Conclusion:**

Rapid, robust, accurate, and precise multislice T1 mapping may be achieved by combining the advantages of a model-based nonlinear inverse reconstruction, radial sampling, parallel imaging, and compressed sensing.

## 1. Introduction

Quantitative T1 mapping is of great interest for application in both research and diagnostics [1]. Fast T1 mapping usually employs an inversion-recovery (IR) look-locker (LL) sequence where RF pulses are continuously applied after inversion with T1 determination in a postprocessing step [2, 3]. Based on the IR LL sequence, recent advances in real-time MRI [4, 5] and model-based reconstructions [6–8] enabled single-shot T1 mapping within a few seconds. The first method [5] defines the IR readout by a series of highly undersampled radial fast low-angle shot (FLASH) images with nonlinear inversion (NLINV) reconstruction [9], while the latter methods reconstruct all parameter maps directly from highly undersampled k-space data, bypassing the intermediate step of image reconstruction completely. Compared to image-space methods such as NLINV, model-based reconstructions [10] allow for a more efficient exploitation of redundancy in the data. Thus, model-based approaches have also been investigated for accelerated T2 mapping [11–13], diffusion [14], flow [15], and MR fingerprinting [16].

In general, quantitative parametric mapping so far focused on single slices, whereas in clinical practice, multislice mapping is highly desirable. For example, it has been recommended to perform myocardial T1 mapping in at least three short-axis sections to capture potential heterogeneity across the left ventricular wall [17]. Previous studies have used a variable flip angle (VFA) approach [18] to acquire volume T1 maps within a clinically acceptable time. While efficient, this method is sensitive to B1 field inhomogeneity and motion might be a problem for abdominal applications [19, 20]. Compared to the VFA approach, the IR LL technique is more tolerant to B1 field inhomogeneities [2, 3]. Early efforts based on the IR LL sequence usually employed segmented Cartesian acquisition schemes which involve a waiting period between the steady state of one segment and the inversion of the next segment. Although different strategies [21, 22] have been proposed to sample the relaxation process more efficiently, it still takes minutes to achieve multislice T1 maps. Therefore, a spiral acquisition with through-time generalized autocalibrating partial parallel acquisitions [20] and the use of spatiotemporal sparsity constraints [23] have been proposed to accelerate volumetric T1 mapping for abdominal applications to about 20 s breath holds. More recently, radial spoke-sequential FLASH with NLINV reconstruction resulted in 5 T1 maps of the human brain within a single IR [24]. However, because all these methods rely on reconstructions in image space, they have to provide a sufficient number of good-quality images during IR in order to allow for subsequent T1 fitting. This condition not only enforces restrictions on the choice of k-space trajectories sampling the entire IR but also renders image-space methods unlikely to fully exploit the inherent redundancy in the data (i.e., about 1000 radial spokes for a 4 s acquisition). This becomes obvious when considering that image-space methods have to reconstruct up to 80 images for a single IR, while all what is needed are only three quantitative maps (i.e., equilibrium magnetization, effective flip angle, and T1).

This study therefore aimed at developing an efficient model-based reconstruction technique which takes advantage of the inherent data redundancy by allowing for simultaneous (i.e., spoke-interleaved) multislice T1 mapping at high spatial resolution. The approach combines a single-shot IR multislice radial FLASH acquisition and a model-based nonlinear inverse reconstruction with joint sparsity constraints. Apart from validations using numerical simulations and experimental phantoms, the performance of the proposed method is demonstrated for studies of the human brain and liver.

## 2. Methods

### 2.1. Single-Shot Multislice Data Acquisition

**Figure 1:**
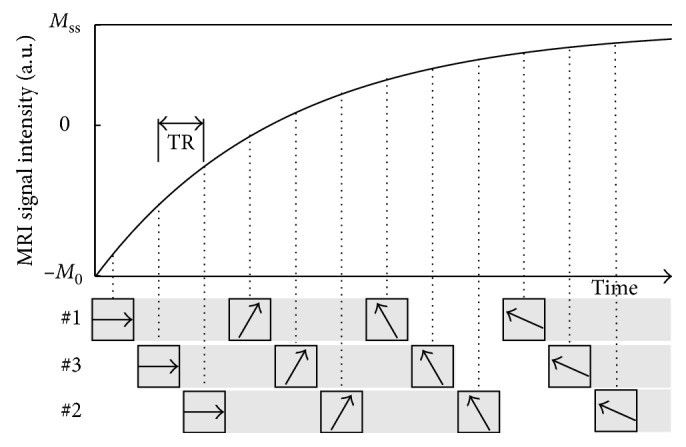
Single-shot inversion-recovery spoke-interleaved radial FLASH acquisition for model-based reconstruction: exemplary scheme for 3 slices. Gray shades indicate that a small number of spokes are binned to reduce the computational (memory) demand.

The data acquisition scheme is demonstrated in Figure 1. Multislice radial k-space data are continuously acquired after a single nonselective inversion pulse using a small-angle golden-angle sampling scheme [25, 26] in a spoke-interleaved manner. The interslice distance is chosen to be at least the slice thickness to avoid cross talk between slices. The relaxation process for each individual section follows the three-parameter model according to the Bloch equations as follows [2, 3]:

(1) $$\begin{array}{c} M_{t_{k}}\left(\vec{r}\right)=M_{\text{ss}}\left(\vec{r}\right)-\left(M_{\text{ss}}\left(\vec{r}\right)+M_{0}\left(\vec{r}\right)\right)\cdot e^{-t_{k}\cdot R_{1}^{*}\left(\vec{r}\right)}, \end{array}$$

where *M*_ss_ represents the steady-state magnetization, *M*_0_ is the equilibrium magnetization, $\vec{r}$ is the position in image space, and *R*_1_^*∗*^ is the effective relaxation rate given by

(2) $$\begin{array}{c} R_{1}^{*}=\frac{1}{T_{1}^{*}}=\frac{1}{T_{1}}-\frac{\text{ln cos}\;\;\alpha }{n_{\text{sl}}\cdot \text{TR}}, \end{array}$$

with TR the repetition time between successive RF pulses as shown in Figure 1, *α* the flip angle, and *n*_sl_ the total number of slices. The sought quantity T1 is then calculated according to the following exact formula after estimation of *M*_ss_, *M*_0_, and *R*_1_^*∗*^:

(3) $$\begin{array}{c} T_{1}=\frac{-n_{\text{sl}}\cdot TR}{\ln\left[1-\left(M_{\text{ss}}/M_{0}\right)\left(1-e^{-n_{\text{sl}}\cdot \text{TR}\cdot R_{1}^{*}}\right)\right]}. \end{array}$$

### 2.2. Model-Based Reconstruction

As described in [8], the signal received from multiple receiver coils can be written as

(4) $$\begin{array}{c} y_{j}\left(t\right)=\int^{}M_{t_{k}}\left(\vec{r}\right)c_{j}\left(\vec{r}\right)e^{-i\vec{r}\vec{k}\left(t\right)}d\vec{r}, \end{array}$$

with *c*_*j*_ the *j*th coil sensitivity map, $\vec{k}\left(t\right)$ the chosen k-space trajectory, *M*_*t*_*k*__ the prescribed model in (1), and *y*_*j*_ the acquired data. Both the parameter maps (*M*_ss_, *M*_0_, and  *R*_1_^*∗*^) and coil sensitivity maps (*c*_1_,…, *c*_*N*_) are directly estimated from the acquired k-space data using a single-step model-based reconstruction, that is,

(5) $$\begin{array}{c} \hat{x}=\underset{x\in D}{\mathrm{argmin}}{\left\|F\left(x\right)-y\right\|}_{2}^{2}+\alpha R\left(x_{\text{p}}\right)+\beta Q\left(x_{\text{c}}\right), \end{array}$$

where *F* is the nonlinear forward model mapping all unknowns *x* to the measured data *y*. *x*_p_ and *x*_c_ represent the parameter maps and the coil sensitivity maps, respectively. *R*(·) is a L1 regularization to exploit joint sparsity in the parameter maps following the ideas of compressed sensing, while *Q*(·) is the Sobolev norm on the coil sensitivities to enforce its intrinsic smoothness. *α* and *β* are the corresponding regularization parameters. *D* is a convex set representing the acceptable domain for the unknowns. Similar to [8], *D* is set to be *R*_1_^*∗*^ ≥ 0 as the relaxation rate should be nonnegative. The nonlinear inverse problem in (5) is solved by the iteratively regularized Gauss–Newton method (IRGNM) [27] where the nonlinear problem is linearized in each Gauss–Newton step and solved by the fast iterative shrinkage-thresholding algorithm [28].

### 2.3. Numerical Simulations

The numerical phantom designed in [8] was used for validations. T1 relaxation times of the phantom are 300 ms, 800 ms, 1500 ms (circular objects), and 2000 ms (background). The k-space data were derived from the analytical Fourier representation of an ellipse assuming an array of four circular receiver coils surrounding the phantom without overlap [29]. The simulations employed the spoke-interleaved multislice IR FLASH sequence with the acquisition parameters as listed in Table 1. Complex white Gaussian noise with a standard deviation of 0.1 was added to the simulated k-space data.

### 2.4. MRI

All MRI measurements were performed on a human 3T MRI system (Magnetom Prisma fit, Siemens Healthineers, Erlangen, Germany). Phantom and brain studies were conducted with a standard 64-channel head coil, while abdominal scans were performed with an 18-element thorax coil in conjunction with 18 elements of the 32-element spine coil. During technical developments, 6 subjects without known illness were recruited among the students of the local university. Written informed consent, according to the recommendations of the local ethics committee, was obtained from all subjects prior to MRI.

The proposed method was experimentally validated with a commercial reference phantom (Diagnostic Sonar LTD, Scotland, UK) consisting of 6 compartments with defined T1 values surrounded by water. Reference T1 maps are taken from the single-slice acquisition whose accuracy and precision have been validated relative to T1 maps calculated from fully sampled data sets [8]. All the acquisition parameters are summarized in Table 1. For IR FLASH, B0 shimming and center-frequency adjustment were performed using the standard procedures provided by the manufacturer. Any residual transverse magnetization was spoiled by random RF phases [30]. Abdominal studies were performed during a brief breath hold.

In the spoke-interleaved multislice acquisition scheme, the RF repetition time increases for any given slice compared to a single-slice measurement (2). Accordingly, the flip angle can also be increased within a certain range to optimize the overall signal-to-noise ratio (SNR). However, with increasing flip angles, T1 accuracy may suffer as the applied correction formula loses validity [3]. Therefore, we varied the flip angles systematically based on experimental phantom studies. An optimal flip angle is then chosen that balances T1 accuracy and SNR for each acquisition. The validations showed that flip angles of 10°, 12°, and 14° turned out to be optimal for 3-slice, 5-slice, and 7-slice acquisitions, respectively. These values were used for all subsequent studies.

Model-based reconstruction techniques in general do not attempt to reconstruct images, and consequently, data binning prior to reconstruction is not necessary. However, a certain degree of temporal discretization may effectively reduce the computational demand without posing too rigid restrictions on probing of the IR. Here, the number of binned spokes was chosen such that the temporal bin size does not exceed 80 ms and T1 accuracy is not compromised [7]. Small golden angles [26] were then adjusted for each data acquisition to ensure a homogeneous coverage of each k-space.

### 2.5. Implementation

At this stage, all data processing was done offline. Multicoil raw data for each slice were first corrected for gradient delays [31] and then compressed to 8 virtual channels using a principle component analysis. A convolution-based gridding [32] without density compensation was used to interpolate the radial samples onto a Cartesian grid on which all successive computations were performed. Gradient delay correction, channel compression, and gridding were done in MATLAB (MathWorks, Natik, MA), while the iterative optimization was implemented in C/CUDA using GeForce GTX TITAN (NVIDIA, Santa Clara, CA).

Regularization parameters *α* and *β* are initially set to 1 and subsequently reduced by a factor of 3 in each Gauss–Newton step. A minimum value of *α* was introduced to control the noise in higher Gauss–Newton steps. The chosen value of *α*_min_ = 0.0015 for applications to the brain was defined by optimizing SNR without compromising quantitative accuracy or delineation of structural details. With similar settings, *α*_min_ = 0.001 was chosen for abdominal studies. Constants in the Sobolev norm were the same as in [8]. As *β*_min_ was insensitive to the final results, no minimum value was set for *β*. Similar to [8], 10 Gauss–Newton steps were used for IRGNM to ensure convergence.

## 3. Results

**Figure 2:**
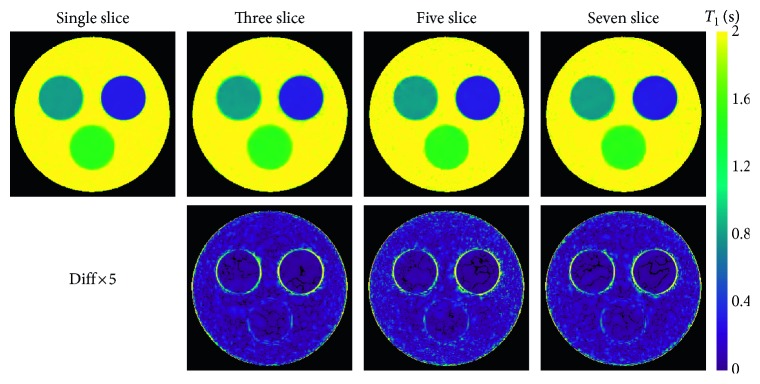
Model-based single-slice and multislice T1 maps 4 s (top) and difference maps relative to the single-slice acquisition for a numerical phantom (bottom; for parameters see Table 1, for quantitative results see Table 2).

Figure 2 shows T1 maps of a numerical phantom obtained by model-based reconstruction from single-slice, 3-slice, 5-slice, and 7-slice acquisitions. The total number of spokes per slice decreased from 1064 to 364, 225, and 156, respectively. Visual inspection of the T1 and difference maps reveals no detectable differences between single-slice and multislice acquisitions. Quantitative ROI analyses in Table 2 confirm the T1 accuracy of all multislice acquisitions, while T1 precision slightly reduces (i.e., standard deviations slightly increase) for increasing number of slices and mostly for shorter T1 values.

**Figure 3:**
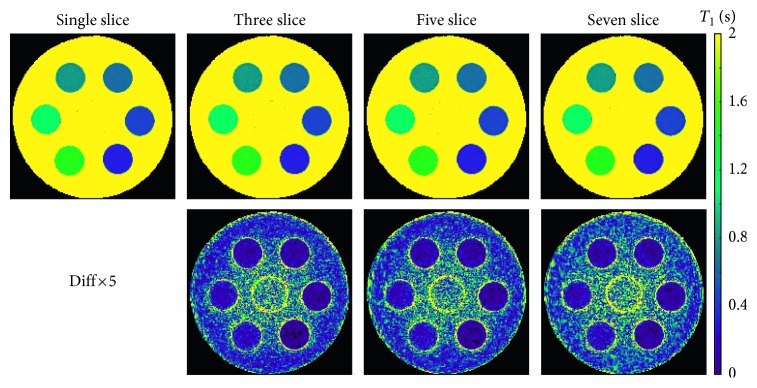
Model-based single-slice and multislice T1 maps 4 s (top) and difference maps relative to the single-slice acquisition for an experimental phantom (bottom; for parameters see Table 1, for quantitative results see Table 3).

Figure 3 and Table 3 offer a similar comparison of single-slice and multislice results for an experimental T1 phantom comprising six tubes with different T1 values. All measurements represent single-shot IR acquisitions with a duration of 4 s. Again, there is no visual difference across T1 and difference maps, while Table 3 confirms excellent T1 accuracy and precision (i.e., small standard deviations) for all measurements and the entire range of T1 values.

**Figure 4:**
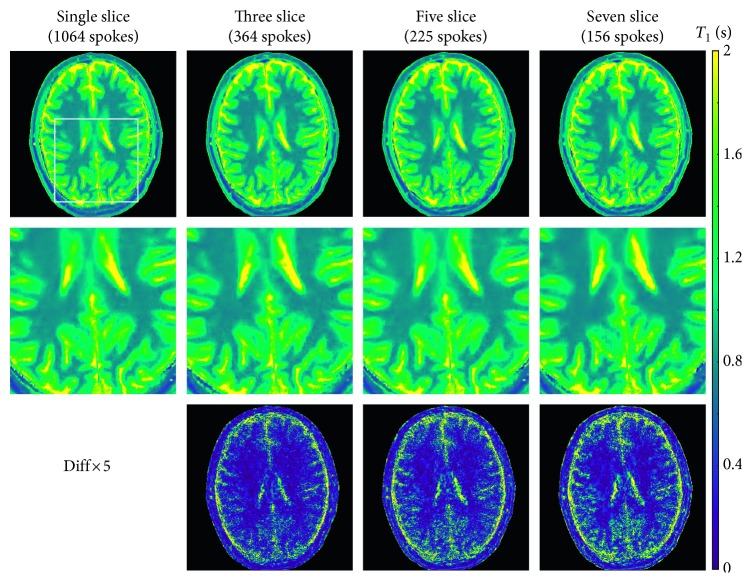
Model-based single-slice and multislice T1 maps 4 s (top), magnified views (middle), and difference maps relative to the single-slice acquisition for the human brain (bottom; for parameters see Table 1, for quantitative results see Table 4).

**Figure 5:**
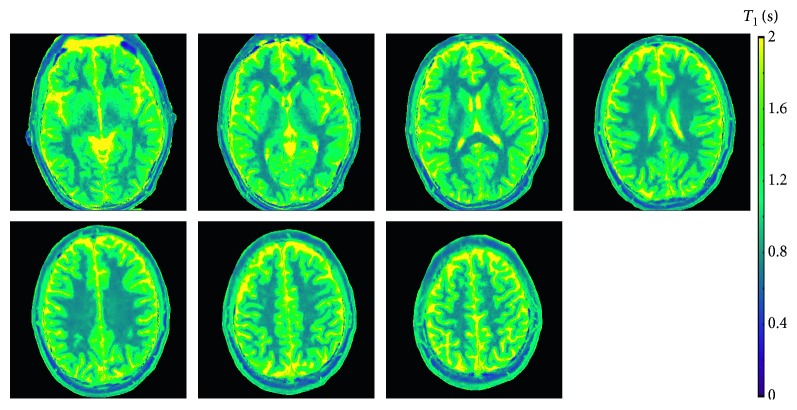
Seven simultaneous T1 maps of the human brain obtained by model-based 7-slice T1 mapping within 4 s (for parameters see Table 1).

**Figure 6:**
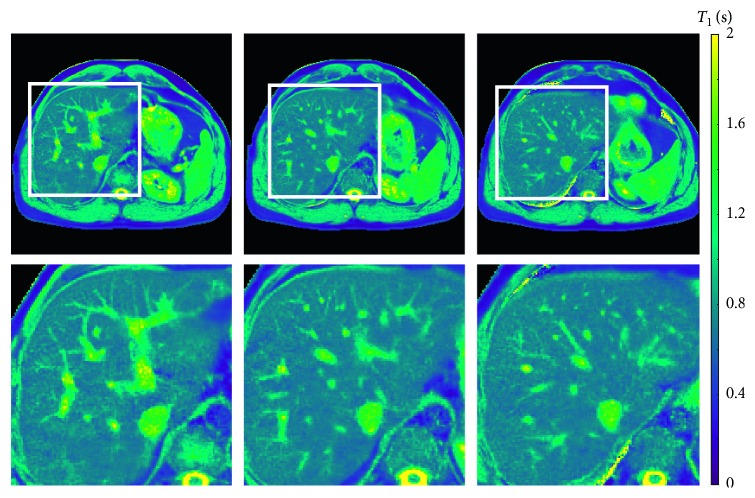
Three simultaneous T1 maps (top) and enlarged views of the human liver obtained by model-based 3-slice T1 mapping within a 4 s breath hold (bottom; for parameters see Table 1).

Figure 4 compares high-resolution T1 maps, enlarged views, and difference maps of the human brain (same section) obtained by single-slice and multislice acquisitions in 4 s. In line with phantom validations, multislice T1 maps are of comparable quality to the single-slice map. This is best demonstrated by the enlarged T1 maps (Figure 4, middle row) and the quantitative analyses in Table 4.  Figure 5 summarizes all 7 maps of a 7-slice T1 mapping experiment using the proposed model-based reconstruction. Similarly, Figure 6 presents the results of a 3-slice T1 mapping study of the liver obtained within a single 4 s breath hold. The magnified views clearly demonstrate good spatial definition of structural details as well as adequate SNR, that is, good precision. The quantitative results yield the regional liver T1 values of 775 ± 34 ms, 783 ± 26 ms, and 766 ± 29 ms (analyzed on regions-of-interests (ROIs) of 144 pixels per T1 map) for the three sections in agreement with literature data of 767 to 812 ms [38, 39].

## 4. Discussion

This work presents a novel multislice T1 mapping technique which combines a single-shot IR spoke-interleaved multislice FLASH acquisition with a sparsity-constrained model-based nonlinear inverse reconstruction. In contrast to mapping techniques based on image space with subsequent fitting, the combined model-based approach effectively exploits the data redundancy in single-slice IR acquisitions. In the present study, this refers to the acquisition of about 1000 radial spokes per single-shot 4-second IR which are available for the reconstruction of only three-parametric maps rather than for up to 80 images (both cases also include all coil sensitivity maps). The present results demonstrate that the radial information available in a single-shot IR acquisition may effectively be used for multislice T1 mapping without compromising resolution, accuracy, and precision as validated by numerical simulations and experimental phantoms.

While 7 simultaneous T1 maps were achievable for the brain, only 3 T1 maps of the liver were presented. This is mainly because of the more pronounced presence of off-resonance contributions (e.g., air-tissue susceptibility differences) within the abdomen which, at higher degrees of undersampling for 5-slice or 7-slice acquisitions, eventually cause residual streaking artifacts. Another influential factor is the need for a higher number of spokes to avoid streaks caused by objects outside the chosen field-of-view, for example, arms lying next to the body [40]. Both effects are also taken care off by sampling the IR with more spokes in a 3-slice acquisition.

Simultaneous multislicing (SMS) is another valuable technique to accelerate multislice imaging in general [41] as well as multislice parametric mapping [42, 43]. For example, a combination of SMS and magnetic resonance fingerprinting achieved three simultaneous T1 maps within 12 s [42]. SMS has also been shown to accomplish 3-slice myocardial T1 mapping [43]. In this respect, the combination of the present model-based reconstruction and SMS would be an interesting next step. It might further increase the number of T1 maps or offer access to even higher spatial resolution.

At this stage, a major limitation of the proposed method is the long offline computational time. This seems to be a general problem for model-based reconstructions (at least for T1 mapping) as all the data have to be kept in memory during iterative optimization. However, several acceleration techniques are under development, including the extension of the current single-GPU version to a multi-GPU implementation or integration of model-based reconstructions into Berkeley Advanced Reconstruction Toolbox [44].

## 5. Conclusion

The proposed method offers rapid and robust single-shot multislice T1 mapping with high accuracy and precision by combining the advantages of model-based nonlinear inverse reconstruction, radial sampling, parallel imaging, and joint sparsity constraints. This novel approach effectively exploits the inherent redundancy of a single-shot spoke-interleaved IR LL experiment. It now warrants clinical evaluations as well as extensions to other applications such as single-shot multislice myocardial T1 mapping.

## Figures and Tables

**Table 1:** Acquisition parameters for model-based multislice T1 mapping.

	Phantom/head				Abdomen	
	1 slice	3 slice	5 slice	7 slice	1 slice	3 slice
Field-of-view (mm^2^)	192 × 192				320 × 320	
Image matrix	384 × 384				256 × 256	
Resolution (mm^2^)	0.5 × 0.5				1.25 × 1.25	
Slice thickness (mm)	4				6	
Repetition time (ms)	3.81				2.98	
Echo time (ms)	2.60				2.20	
Bandwidth (Hz pixel^−1^)	725				1420	
Acquisition time (s)	4.0				4.0	
Flip angle (degree)	6	10	12	14	6	10
Spokes per slice	1064	364	225	156	1344	456
Binned spokes	21	7	4	3	21	7
Golden angle (degree)	20.89	38.98	68.75	68.75	20.89	38.98

**Table 2:** T1 relaxation times (ms, mean ± SD) for a numerical phantom (Figure 2).

Tube	#1	#2	#3	Background
True T1	300	800	1500	2000
1 slice	301 ± 3	800 ± 5	1501 ± 11	2001 ± 12
3 slice	302 ± 4	799 ± 9	1500 ± 22	1997 ± 24
5 slice	300 ± 6	800 ± 10	1498 ± 22	2001 ± 24
7 slice	300 ± 9	801 ± 13	1497 ± 21	2002 ± 27

**Table 3:** T1 relaxation times (ms, mean ± SD) for an experimental phantom (Figure 3).

Tube	#1	#2	#3	#4	#5	#6
Reference^1^	309 ± 4	450 ± 6	622 ± 10	800 ± 11	1158 ± 23	1478 ± 35
3 slice	312 ± 6	455 ± 9	628 ± 11	805 ± 16	1165 ± 27	1483 ± 26
5 slice	313 ± 10	456 ± 10	630 ± 11	810 ± 14	1168 ± 27	1483 ± 33
7 slice	315 ± 7	457 ± 11	633 ± 11	810 ± 10	1158 ± 17	1471 ± 28

^1^Taken from [8].

**Table 4:** T1 relaxation times (ms, mean ± SD) of the human brain.

Brain	Frontal WM	Occipital WM	Frontal GM	Occipital GM
ROI (pixels)	165	135	63	64
Reference^1^	719 ± 17	768 ± 23	1330 ± 58	1389 ± 58
3 slice	711 ± 27	764 ± 26	1359 ± 65	1394 ± 69
5 slice	726 ± 24	775 ± 31	1366 ± 62	1399 ± 87
7 slice	728 ± 28	768 ± 30	1332 ± 86	1403 ± 70
Literature (reference)	699 to 985 [33–36]	758 to 940 [34, 36, 37]	1209 to 1322 [33, 37]	1283 [36]

^1^Taken from [8].

## Data Availability

The data used to support the findings of this study are available from the corresponding author upon request.

## References

[1] Cheng H., Stikov N., Ghugre N. (2012). Practical medical applications of quantitative MR relaxometry. *Journal of Magnetic Resonance Imaging*.

[2] Look D. C., Locker D. R. (1970). Time saving in measurement of NMR and EPR relaxation times. *Review of Scientific Instruments*.

[3] Deichmann R., Haase A. (1992). Quantification of T1 values by snapshot-FLASH NMR imaging. *Journal of Magnetic Resonance*.

[4] Uecker M., Zhang S., Voit D., Karaus A., Merboldt K. D., Frahm J. (2010). Real-time MRI at a resolution of 20 ms. *NMR Biomedicine*.

[5] Wang X., Joseph A. A., Kalentev O. (2016). High-resolution myocardial T1 mapping using single-shot inversion-recovery fast low-angle shot MRI with radial undersampling and iterative reconstruction. *British Journal of Radiology*.

[6] Tran-Gia J., Wech T., Bley T., Köstler H. (2015). Model-based acceleration of look-locker T1 mapping. *PLoS One*.

[7] Roeloffs V., Wang X., Sumpf T., Untenberger M., Voit D., Frahm J. (2016). Model-based reconstruction for T1 mapping using single-shot inversion recovery radial FLASH. *International Journal of Imaging Systems and Technology*.

[8] Wang X., Roeloffs V., Klosowski J. (2018). Model-based T1 mapping with sparsity constraints using single-shot inversion-recovery radial FLASH. *Magnetic Resonance in Medicine*.

[9] Uecker M., Hohage T., Block K. T., Frahm J. (2008). Image reconstruction by regularized nonlinear inversion–joint estimation of coil sensitivities and image content. *Magnetic Resonance in Medicine*.

[10] Fessler J. A. (2010). Model-based image reconstruction for MRI. *IEEE Signal Processing Magazine*.

[11] Block K. T., Uecker M., Frahm J. (2009). Model-based iterative reconstruction for radial fast spin-echo MRI. *IEEE Transactions on Medical Imaging*.

[12] Sumpf T., Uecker M., Boretius S., Frahm J. (2011). Model-based nonlinear inverse reconstruction for T2 mapping using highly undersampled spin-echo MRI. *Journal of Magnetic Resonance Imaging*.

[13] Zhao B., Lam F., Liang Z. P. (2014). Model-based MR parameter mapping with sparsity constraints: parameter estimation and performance bounds. *IEEE Transactions on Medical Imaging*.

[14] Knoll F., Raya J. G., Halloran R. O. (2015). A model-based reconstruction for undersampled radial spin-echo DTI with variational penalties on the diffusion tensor. *NMR Biomedicine*.

[15] Tan Z., Roeloffs V., Voit D. (2017). Model-based reconstruction for real-time phase-contrast flow MRI: improved spatiotemporal accuracy. *Magnetic Resonance in Medicine*.

[16] Zhao B., Setsompop K., Ye H., Cauley S. F., Wald L. L. (2016). Maximum likelihood reconstruction for magnetic resonance fingerprinting. *IEEE Transactions on Medical Imaging*.

[17] Moon J. C., Messroghli D. R., Kellman P. (2013). Myocardial T1 mapping and extracellular volume quantification: a Society for Cardiovascular Magnetic Resonance (SCMR) and CMR Working Group of the European Society of Cardiology consensus statement. *Journal of Cardiovascular Magnetic Resonance*.

[18] Cheng H. L., Wright G. A. (2006). Rapid high-resolution T1 mapping by variable flip angles: accurate and precise measurements in the presence of radiofrequency field inhomogeneity. *Magnetic Resonance in Medicine*.

[19] Treier R., Steingoetter A., Fried M., Schwizer W., Boesiger P. (2007). Optimized and combined T1 and B1 mapping technique for fast and accurate T1 quantification in contrast-enhanced abdominal MRI. *Magnetic Resonance in Medicine*.

[20] Chen Y., Lee G. R., Aandal G. (2016). Rapid volumetric T1 mapping of the abdomen using three-dimensional through-time spiral GRAPPA. *Magnetic Resonance in Medicine*.

[21] Shah N. J., Zaitsev M., Steinhoff S., Zilles K. (2001). A new method for fast multislice T1 mapping. *NeuroImage*.

[22] Deichmann R. (2005). Fast high-resolution T1 mapping of the human brain. *Magnetic Resonance in Medicine*.

[23] Lugauer F., Wetzl J., Forman C. (2018). Single-breath-hold abdominal T1 mapping using 3D Cartesian look-locker with spatiotemporal sparsity constraints. *MAGMA*.

[24] Wang X., Roeloffs V., Merboldt K. D., Voit D., Schätz S., Frahm J. (2015). Singleshot multi-slice T1 mapping at high spatial resolution–inversion recovery FLASH with radial undersampling and iterative reconstruction. *Open Medical Imaging Journal*.

[25] Winkelmann S., Schaeffter T., Koehler T., Eggers H., Doessel O. (2007). An optimal radial profile order based on the Golden Ratio for time-resolved MRI. *IEEE Transactions on Medical Imaging*.

[26] Wundrak S., Paul J., Ulrici J. (2016). Golden ratio sparse MRI using tiny golden angles. *Magnetic Resonance in Medicine*.

[27] Bakushinsky A. B., Kokurin M. Y. (2004). *Iterative Methods for Approximate Solution of Inverse Problems*.

[28] Beck A., Teboulle M. (2009). A fast iterative shrinkage-thresholding algorithm for linear inverse problems. *SIAM Journal on Imaging Sciences*.

[29] Guerquin-Kern M., Lejeune L., Pruessmann K. P., Unser M. (2012). Realistic analytical phantoms for parallel magnetic resonance imaging. *IEEE Transactions on Medical Imaging*.

[30] Roeloffs V., Voit D., Frahm J. (2015). Spoiling without additional gradients: radial FLASH MRI with randomized radiofrequency phases. *Magnetic Resonance in Medicine*.

[31] Block K. T., Uecker M. Simple method for adaptive gradient-delay compensation in radial MRI.

[32] Wajer F., Pruessmann K. P. Major speedup of reconstruction for sensitivity encoding with arbitrary trajectories.

[33] Lu H., Nagae-Poetscher L. M., Golay X., Lin D., Pomper M., Van Zijl P. C. M. (2005). Routine clinical brain MRI sequences for use at 3.0 tesla. *Journal of Magnetic Resonance Imaging*.

[34] Gelman N., Ewing J. R., Gorell J. M., Spickler E. M., Solomon E. G. (2001). Interregional variation of longitudinal relaxation rates in human brain at 3.0 T: relation to estimated iron and water contents. *Magnetic Resonance in Medicine*.

[35] Zhu D. C., Penn R. D. (2005). Full-brain T1 mapping through inversion recovery fast spin echo imaging with time-efficient slice ordering. *Magnetic Resonance in Medicine*.

[36] Preibisch C., Deichmann R. (2009). Influence of RF Spoiling on the stability and accuracy of T1 mapping based on spoiled FLASH with varying flip angles. *Magnetic Resonance in Medicine*.

[37] Wansapura J. P., Holland S. K., Dunn R. S., Ball W. S. (1999). NMR relaxation times in the human brain at 3.0 tesla. *Journal of Magnetic Resonance Imaging*.

[38] Haimerl M., Verloh N., Zeman F. (2013). Assessment of clinical signs of liver cirrhosis using T1 mapping on Gd-EOB-DTPA-enhanced 3T MRI. *PLoS One*.

[39] de Bazelaire C. M., Duhamel G. D., Rofsky N. M., Alsop D. C. (2004). MR imaging relaxation times of abdominal and pelvic tissues measured in vivo at 3.0 T: preliminary results. *Radiology*.

[40] Block K. T., Chandarana H., Milla S. (2014). Towards routine clinical use of radial stack-of-stars 3D gradient-echo sequences for reducing motion sensitivity. *Journal of the Korean Society of Magnetic Resonance in Medicine*.

[41] Barth M., Breuer F., Koopmans P. J., Norris D. G., Poser B. A. (2016). Simultaneous multislice (SMS) imaging techniques. *Magnetic Resonance in Medicine*.

[42] Ye H., Cauley S., Gagoski B. (2017). Simultaneous multislice magnetic resonance fingerprinting (SMS-MRF) with direct-spiral slice-grappa (ds-sg) reconstruction. *Magnetic Resonance in Medicine*.

[43] Weingärtner S., Moeller S., Schmitter S. (2017). Simultaneous multislice imaging for native myocardial t1 mapping: improved spatial coverage in a single breath-hold. *Magnetic Resonance in Medicine*.

[44] Uecker M., Ong F., Tamir J. Berkeley advanced reconstruction toolbox.

